# Development of Two Barthel Index-Based Supplementary Scales for Patients with Stroke

**DOI:** 10.1371/journal.pone.0110494

**Published:** 2014-10-20

**Authors:** Ya-Chen Lee, Sheng-Shiung Chen, Chia-Lin Koh, I-Ping Hsueh, Kai-Ping Yao, Ching-Lin Hsieh

**Affiliations:** 1 School of Occupational Therapy, College of Medicine, National Taiwan University, Taipei, Taiwan; 2 Department of Physical Medicine and Rehabilitation, E-Da Hospital/I-Shou University, Kaohsiung, Taiwan; 3 School of Occupational Therapy, College of Medicine, National Taiwan University, Taipei, Taiwan; 4 Department of Physical Medicine and Rehabilitation, National Taiwan University Hospital, Zhongzheng District, Taipei, Taiwan; 5 Department of Psychology, College of Science, National Taiwan University, Taipei, Taiwan; Shanghai Mental Health Center, Shanghai Jiao Tong University School of Medicine, China

## Abstract

**Background:**

The Barthel Index (BI) assesses actual performance of activities of daily living (ADL). However, comprehensive assessment of ADL functions should include two other constructs: self-perceived difficulty and ability.

**Objective:**

The aims of this study were to develop two BI-based Supplementary Scales (BI-SS), namely, the Self-perceived Difficulty Scale and the Ability Scale, and to examine the construct validity of the BI-SS in patients with stroke.

**Method:**

The BI-SS was first developed by consultation with experts and then tested on patients to confirm the clarity and feasibility of administration. A total of 306 participants participated in the construct validity study. Construct validity was investigated using Mokken scale analysis and analyzing associations between scales. The agreement between each pair of the scales’ scores was further examined.

**Results:**

The Self-perceived Difficulty Scale consisted of 10 items, and the Ability Scale included 8 items (excluding both bladder and bowel control items). Items in each individual scale were unidimensional (*H*≥0.5). The scores of the Self-perceived Difficulty and Ability Scales were highly correlated with those of the BI (rho = 0.78 and 0.90, respectively). The scores of the two BI-SS scales and BI were significantly different from each other (p<.001). These results indicate that both BI-SS scales assessed unique constructs.

**Conclusions:**

The BI-SS had overall good construct validity in patients with stroke. The BI-SS can be used as supplementary scales for the BI to comprehensively assess patients’ ADL functions in order to identify patients’ difficulties in performing ADL tasks, plan intervention strategies, and assess outcomes.

## Introduction

Stroke is the leading cause of disability or dependence in activities of daily living (ADL) among the elderly [1]–[3]. Increasing independence in ADL is often a central aim of stroke management. Assessing a patient’s ADL functions enable clinicians to set reasonable treatment goals, to make appropriate discharge arrangements, and to anticipate the need for community support [2], [4].Thus, ADL measures have been widely used for clinical decision making, treatment planning, and outcome measurement.

There are at least three different constructs for ADL measures: actual performance, self-perceived difficulty, and ability [5]–[9]. Each construct has unique characteristics and provides unique information for users. Actual performance refers to what a person actually does in his/her daily environment and is similar in concept to the qualifier of “performance” in the International Classification of Functioning, Disability and Health (ICF) [8], [10]–[14]. Assessing actual performance can assist users in identifying an individual’s level of dependence in ADL in real life situations [5], [15]. Ability describes a person’s ability to execute an ADL task in a standardized, controlled context and is similar in concept to the qualifier of “capacity” in the ICF [5], [6], [10], [14]. Assessing ADL ability provides concrete/objective information about each ADL task that an individual is physically capable (or incapable) of doing [16]. Self-perceived difficulty defines the difficulty level that a person subjectively perceives when performing ADL without assistance in daily life [5], [9], [17]. Assessing self-perceived difficulty in performing ADL is useful in identifying an individual’s need for assistance and is in line with a patient-centered approach, which recently has been strongly advocated [5], [18].

Because the three ADL constructs differ in concept and clinical utility, assessing all three constructs simultaneously helps users comprehensively understand an individual’s ADL functions. For example, a patient may be capable of going to the toilet by him/herself in a standardized context, but he/she might need assistance in his/her daily life because of the inaccessible condition (e.g., a narrow door to the bathroom) in his/her living environment. On the other hand, although the patient may not be able to do an ADL task in a standardized context, he/she may accomplish it at home through home modification or the use of assistive devices. In addition, patients might report difficulty in performing ADL in spite of being fully able and actually independent in real life. Thus, assessing the three ADL constructs simultaneously will improve the efficacy of stroke management and related research.

To our knowledge, no existing ADL measures assess all three ADL constructs simultaneously. Among the ADL measures, the Barthel Index (BI) has been widely used to assess stroke patients’ actual performance on ADL functions in both clinical and research settings due to its ease of administration and sound psychometric properties [5], [19]–[21]. Thus, the BI has been adopted by the British Geriatric Society and the Royal College of General Practitioners as the recommended scale for assessment of ADL [22]. However, the BI does not assess the other two constructs (i.e., self-perceived difficulty and ability). Thus, the purposes of this study were (1) to develop two supplementary scales (Self-perceived Difficulty Scale and Ability Scale) for the BI, the BI-based Supplementary Scales (BI-SS), in order to comprehensively assess ADL functions; and (2) to examine the construct validity of the BI-SS, which is critical for differentiating the three ADL constructs, in patients with stroke.

## Methods

### Phase 1: Development of the BI-SS

The development process had two stages:

#### Stage one: Consultation with experts to determine the response categories, modes of administration, and administrative instructions of the BI-SS

Two meetings of the expert panel were held in stage one. The panels consisted of 2 senior occupational therapists, 2 psychometricians, and 3 researchers in the field of occupational therapy. The main purpose of the first meeting was to decide response categories and modes of administration (e.g., face-to-face interview or performance observation) for the Self-perceived Difficulty Scale and the Ability Scale, respectively. The definitions of ADL constructs were explained and the 10 items of the BI were provided to the panel members to act as reference items for the Self-perceived Difficulty Scale and the Ability Scale. In the second meeting, the expert panel developed the standardized administrative instructions for each item of the BI-SS based on the modes of administration determined from the first meeting. In addition, the panel members determined whether any tools or materials would be needed for assessment. All 7 of the panel members attended and participated in these two meetings. It was considered that consensus was achieved when at least 80% of the panel members indicated agreement with a proposal.

#### Stage 2: A pilot test of the BI-SS in patients with stroke

A pilot test was conducted with the patients to examine the clarity of the administrative instructions and the feasibility of administration of the BI-SS. We tried to recruit participants having characteristics similar to those of the target patients. All procedures were carried out by the first author in an assessment room. Participants were individually tested and encouraged to identify any administrative instructions or response categories that seemed difficult to understand or ambiguous to them. The comments were reviewed and changes were made after 4 participants were tested. This process (testing and revisions) was repeated until no more substantial comments were made.

### Phase 2: Examination of the construct validity of the BI-SS

#### Subjects

Patients undergoing outpatient or inpatient rehabilitation were recruited from 7 rehabilitation departments in Taiwan (including northern (4 hospitals), central (2 hospitals), and southern (1 hospital) parts of Taiwan) between January 2011 and August 2012.

Participants were included in the study if they met the following criteria: (1) diagnosis (*International Classification of Disease, Ninth Revision, Clinical Modification codes*) of cerebral hemorrhage (431), cerebral infarction (434), or other (430, 432, 433, 436, 437); and (2) ability to follow instructions. In addition, we excluded patients with any co-morbidity (e.g., dementia, Parkinsonism, limb amputation, or spinal cord injury) that might otherwise affect the patient’s performance on ADL. All participants gave informed consent prior to their inclusion in the study. Demographic characteristics and information on co-morbidities were collected from their medical records.

#### Ethics statement

This study was approved by the Research Ethics Committee Office of E-DA Hospital and the Institutional Review Board of Kaohsiung Medical University Chung-Ho Memorial Hospital.

#### Procedure

Each participant was assessed with the BI-SS and BI once by one of the two trained raters in an assessment room. The BI was administered to the participants via face-to-face interview with the original scoring criteria [23].

Prior to the study, the raters (independent of the expert panel) familiarized themselves with the BI-SS and BI. Both raters studied the user manual of the BI-SS and BI and received 2 hours of training on the administration of the BI-SS and BI. At the end of the training, both raters individually administered the BI-SS and BI to two patients while the first author observed and scored the patients at the same time. The raters’ scoring results were checked by the first author. Any discrepancies in score results were discussed to ensure that the raters were thoroughly familiar with the standardized process of administration and scoring criteria.

#### Data analysis

We validated the construct validity of the BI-SS by examining the unidimensionality and convergent validity of the BI-SS.

*Unidimensionality.* We examined the unidimensionality of each scale of the BI-SS individually using Mokken scale analysis with the MSP5.0 computer program [3]. Mokken scale analysis is a nonparametric item response theory (IRT). The model of monotone homogeneity (MH) of Mokken scale analysis examines the accuracy of ordering of between persons’ raw sum scores on a measure to determine undimensionality [3], [22]. The MH model of the Mokken scale was used because it is believed to exemplify the simplest form of unidimensionality [24], [25]. Other parametric IRT models, such as the Rasch model, further require a parametric functional form of the item response function (IRF) [25], [26]. However, with rigorous assumptions, the Rasch model tends to exclude items that do fit the unidimensionality assumption (e.g., the Mokken model’s expectations) but not the parametric IRF form assumption. Thus, the Mokken model is likely to include more items from a pool of items in a scale while still holding the essential of unidimensionality [24].

The MH model has three assumptions: (1) items form a unidimensional scale (measuring the same construct; e.g., ADL ability); (2) item scores are locally independent (e.g., the scores on a given set of items are stochastically independent of each other within a group of persons with the same level of ADL ability); and (3) the item response function for each item is a steadily increasing function of the latent trait which means that patients with a higher level of ADL function would have a higher probability of scoring higher for an item that fits MH [24], [27]. Given a set of items (e.g., 8 items of the Ability Scale) that satisfies the assumptions of the MH model, then unidimensionality will hold, and it is justified to sum the score of each items to create a total score to represent the construct of interest (e.g., ADL ability) [25], [28].

The fit of the MH model was evaluated by calculating the scalability coefficient *H* for each of the individual items *i (Hi)* and for the entire measure (*H*). The *Hi* value was evaluated to determine whether an item was coherent enough to be included in a unidimensional scale. In general, all *Hi* in a unidimensional scale should be ≥0.3 [27]. Thus, we removed items from the BI-SS that had a *Hi* below 0.3. The *H* value is a global indicator of the degree to which participants can be accurately ordered on the underlying construct by means of their sum scores. Higher values of *H* indicate fewer violations of the assumption and a better scale [3], [24], [25]. Therefore, unidimensionality was considered to be strongly supported if *H*≥0.5 [3].

*Convergent validity.* Spearman’s rho correlation coefficient was used to examine the association between the BI-SS and the BI to determine the convergent validity of the BI-SS. A rho value ≥0.75 was considered high, 0.40–0.74 moderate, and ≤0.39 low [29]. We expected that the three scales would have moderate to high associations with each other.

We further examined the agreement between each pair of scores of the three scales (i.e., BI and BI-SS) to confirm that they were distinguished scales. First, the Wilcoxon signed rank test was used to examine whether the scores of the three scales were significantly differently from each other. The Wilcoxon signed rank test is a nonparametric statistical hypothesis test used to investigate the difference between the magnitudes of paired (dependent) observations (i.e., the BI and BI-SS in this study) [30]. Second, the minimal important difference (MID; also known as the minimal clinically importance difference [31], [32]) of the BI (i.e., 1.85 points) [33] was used as a threshold to present a meaningful difference in the responses of each participant between the scales. The proportions of the patients whose response differences between each pair of scales exceeded 1.85 points were calculated. To visualize the magnitude of response differences and the degree of agreement between scales, Bland-Altman plots with 95% limits of agreement (LOA) [34] were also plotted. The LOA provided insight into the amount of variation between scales. The agreement and variation of each patient’s responses to each pair of the three scales could also be seen on the plot. The range of difference was largely defined by interval between the upper bound and the lower bound of the 95% LOA (d±1.96×SD), where d represents the mean differences of the each pair of scores and SD represents the standard deviation of differences [34]. If a pair of scale assesses the same construct, then the pair of scores will agree very closely and the ranges of differences between both scales will be small. Third, we compared the numbers of patients with the lowest and highest scores in the BI against the two BI-SS scales.

## Results

### Phase 1: Development of the BI-SS

#### Stage one: Consultation with experts to determine the response categories, modes of administration, and administrative instructions of the BI-SS

Based on the results of expert panel discussions, each of the 10 items of the Self-perceived Difficulty Scale used 3 response categories ranging from 0 (with much difficulty), 1 (with some difficulty), and 2 (without any difficulty), with a total score of 20 (Appendix S1 in File S1). The higher the score, the lower the patient’s self-perceived difficulty in performing ADL.

Regarding the mode of administration, the face-to-face interviews method was decided for the Self-perceived Difficulty Scale. Thus, the Self-perceived Difficulty Scale was administered by asking patients to respond to questions such as “How much difficulty do you have in performing grooming?” Because self-perceived difficulty is based on a patient’s own perception, it will be valid only if the responses are from the patient him/herself.

Two items (bowel and bladder control) were removed from the Ability Scale due to their infeasibility and non-practicality to be assessed in clinical settings, leaving only eight items. The items of the Ability Scale had 3 or 4 response categories. For example, ‘grooming’ could be rated 0 (unable to perform), 1 (able to complete partially), or 2 (able to complete), while ‘transferring’ could be rated 0 (unable to perform), 1 (barely able to complete), 2 (almost able to complete), or 3 (able to complete) (Appendix S1 in File S1). The total score ranged from 0 to 18, with higher scores implying a higher level of ability to carry out the ADL. Further detailed instructions for scoring the Ability Scale can be found in Appendix S1 in File S1.

Regarding the mode of administration, observation-based testing was used for the Ability Scale. In addition, the panel members recommended that the Ability Scale be assessed in a standardized context (e.g., an assessment room without distractors such as physical obstacles or other people) to eliminate the varying impacts of different contexts on the performance of a patient. Furthermore, panel members decided on the tools/materials to be used for assessing the items of feeding, grooming, dressing, and bathing in the Ability Scale: chopsticks, spoons, a bowl, a brush, toothpaste, clothes, and towels. Thus, the Ability Scale was assessed by observing patients as they carried out a specific ADL task, such as “put the jacket on and zip it up”. Then the rater rated the patient’s level of ability in doing this task.

#### Stage two: A pilot test of the BI-SS in patients with stroke

A total of 12 patients participated in the stage two of pilot testing to confirm the administrative instructions and feasibility of the BI-SS. Three rounds of testing were carried out. In the first and second rounds of testing, patients gave comments on the ambiguous wordings of instructions for, e.g., the eating task with chopsticks and the dressing task. Thus, the revisions were made accordingly. In the third round of testing, no substantial changes were suggested. The final version of standardized administrative instructions for each item of the BI-SS was clear, and the modes of administration and response categories were understandable to the patients. Thus, no further testing was conducted. In addition, on the basis of the third round of testing, the time required to complete the BI-SS was about 15 minutes.

### Phase 2: Examination of the construct validity of the BI-SS

A total of 306 participants participated in this study. Their mean age was about 61 (SD = 13.8) years, and 64.1% of the patients were male. Of these participants, 62.1% of stroke was caused by cerebral infarction. The scores of the BI ranged from 0 to 20 (i.e., the full possible score range), indicating that the participants had a wide range of ADL function. Further characteristics of the participants are shown in Table 1.

**Table 1:** Characteristics of the participants (n = 306).

Characteristic	
Gender (male/female) (%)	196/110 (64.1%/35.9%)
Age, mean (SD)	61.82 (13.8)
Days after onset, median (1^st^ quartile – 3^rd^ quartile)	77.5 (28–416)
Diagnosis, *n*	
Cerebral hemorrhage (%)	116 (37.9%)
Cerebral infarction (%)	190 (62.1%)
Side of hemiplegia, *n*	
Right	176
Left	120
Bilateral	10
BI score, median (1^st^ quartile – 3^rd^ quartile)	13.0 (8–17)
Self-perceived Scale score, median (1^st^ quartile – 3^rd^ quartile)	12.0 (7–17)
Ability Scale score, median (1^st^ quartile – 3^rd^ quartile)	12.5 (8–16)

Table 2 summarizes the results of the evaluation of the Mokken scale analysis for the BI-SS. The scalability coefficients *Hi* for the items in relation to each individual scale were all above 0.3 (ranging from 0.49 to 0.82). In addition, scalability coefficients *H* of the 10 items of the Self-perceived Difficulty Scale and 8 items of the Ability Scale were greater than 0.5 (*H*≥0.63), strongly supporting the unidimensionality of the items of each scale.

**Table 2:** Results of Mokken scale analysis on the items of the BI-SS (n = 306).

		Self-perceived Difficulty Scale	Ability Scale
Item		*Hi*	*Hi*
1	Feeding	0.49	0.59
2	Grooming	0.55	0.61
3	Dressing	0.67	0.69
4	Bathing	0.64	0.71
5	Bowels	0.68	-
6	Bladder	0.62	-
7	Toilet use	0.71	0.82
8	Transfer	0.66	0.80
9	Mobility	0.63	0.76
10	Stairs	0.62	0.79
Scale *H*		0.63	0.75

**Figure 1:**
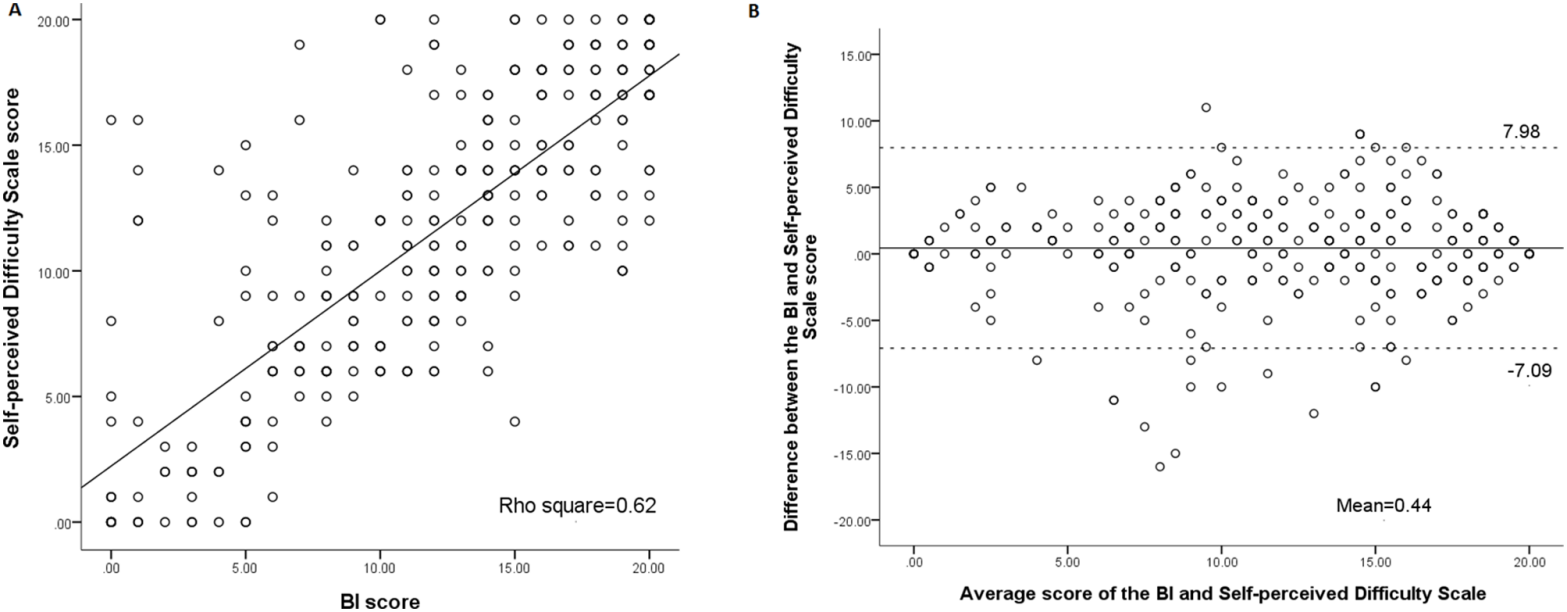
Correlation (A) and Bland-Altman plot (B) for the BI and Self-perceived Difficulty Scale. Bland-Altman method for plotting the scores of the difference between the BI and Self-perceived Difficulty Scale. The 2 dashed lines define the limits of agreement (mean of difference ±1.96 SD).

**Figure 2:**
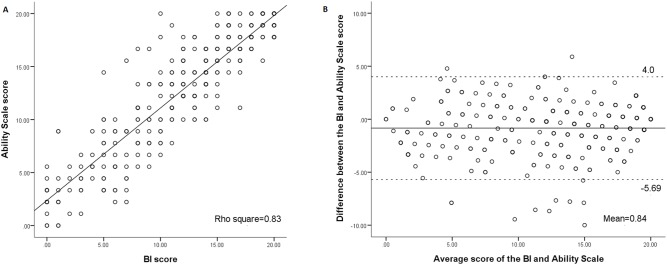
Correlation (A) and Bland-Altman plot (B) for the BI and Ability Scale. The Ability Scale scores were 0•20 transformed scores.

**Figure 3:**
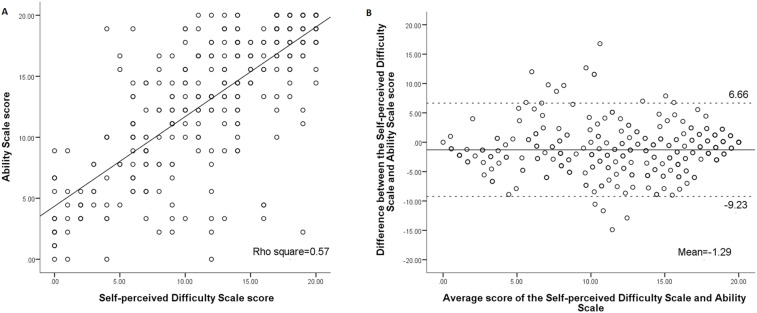
Correlation (A) and Bland-Altman plot (B) for the Self-perceived Difficulty Scale and Ability Scale. The Ability Scale scores were 0•20 transformed scores.

Figures 1, 2, and 3 show association and agreement between scores of the three scales. The BI was highly correlated with the Self-perceived Difficulty Scale (rho = 0.78) and the Ability Scale (rho = 0.90), respectively. The Self-perceived Difficulty Scale was highly correlated with the Ability Scale (rho = 0.75).

In order to further compare the three scales, the scores of the Ability Scale were linearly transformed into the same score ranges as those of the other two scales (0–20). First, the Wilcoxon signed rank test showed that the scores of the three scales were significantly different from each other (p<.001) (Table 3). Second, the proportion of the patients whose difference between two scales was beyond 1.85 points (MID) were 60.1% for the BI and Self-perceived Difficulty Scale, 41.8% for the BI and Ability Scale, and 61.4% for the Self-perceived Difficulty Scale and Ability Scale. The ranges of differences between each pair of the three scales are shown in the Bland-Altman plots (Figs. 1, 2, 3). The width of LOA was 15.1 for the BI and Self-perceived Difficulty Scale (75.5% of the maximal score range, 20), 9.7 for the BI and Ability Scale (48.5% of the maximal score range, 20), and 15.9 for the Self-perceived Difficulty Scale and Ability Scale (79.5% of the maximal score range, 20).

**Table 3:** The results of the agreement between the paired scales and the numbers of participants whose difference between 2 scales was beyond 1.85 points.

	Meandifference	Wilcoxon Z(p-value)	Number of participants with difference between 2 scales beyond 1.85 points (%)
BI score vs. Self-perceivedDifficulty Scale score	0.44	−3.6 (<.001)	184 (60.1%)
BI score vs. AbilityScale score	−0.84	−5.3 (<.001)	128 (41.8%)
Self-perceived DifficultyScale score vs. AbilityScale score	−1.29	−6.6 (<.001)	188 (61.4%)

Third, to further present the differences in the patients’ scores on the three scales, we compared the numbers of patients who obtained extreme scores on these scales. A total of 17 patients scored 0 (with much difficulty) on the Self-perceived Difficulty Scale, but more than half (n = 9) of these 17 patients obtained total scores >0 on the BI. Twenty-one patients scored 20 (without any difficulty) on the Self-perceived Difficulty Scale, but nearly half (n = 10) of these 21 patients obtained total scores <20 on the BI. A total of 32 patients scored the highest possible score on the Ability Scale, but about 60% (n = 19) of these 32 patients did not obtain the highest possible score on the BI. A total of 4 patients scored the lowest possible score on the Ability Scale, but 75% (n = 3) of these patients did not obtain the lowest possible score on the Self-perceived Difficulty Scale.

## Discussion

The aim of this study was to develop a supplementary measure based on the original BI, the BI-SS, in order to comprehensively assess ADL functions. Analyzed with the MH model of Mokken scale analysis, our results showed that the unidimensionality of the two ADL construct scales were strong (*H*≥0.63). The results indicated that the 10 items of the Self-perceived Difficulty Scale assessed a single dimension, as did the 8 items of the Ability Scale. Because the items of each scale of BI-SS assessed the same dimension, the results supported summating the raw score of each item in each individual scale to create a total score for their respective scales to represent patients’ level of function on self-perceived difficulty and ability.

We used the original BI as a criterion to examine the convergent validity of both the Self-perceived Difficulty Scale and the Ability Scale. Our results showed a high degree of correlation between the original BI and the two scales (rho = 0.78 and 0.90, respectively), indicating that both constructs measured by the BI-SS, self-perceived difficulty and ability, were highly related to the actual performance construct in patients with stroke. The results confirm our hypotheses and support the convergent validity of the BI-SS in patients with stroke. Combining the results of sufficient unidimensionality and convergent validity of the BI-SS, the construct validity of the BI-SS is highly supported.

Although the associations between any pairs of the three scales were high, the other results showed that the three scales were different from each other. First, the unexplained variance between the scales was substantial (i.e., 19.1% unexplained variance existing between the BI and Ability Scale, 38% between the BI and Self-perceived Difficulty Scale, and 43.8% between the Self-perceived Difficulty Scale and Ability Scale). Second, the range of disagreement between scales was widely distributed. The LOAs revealed large variations between scales. Particularly, about half (41.861.4%) of the patients had important differences (>1.85) between scales. Third, about half to three quarters (47.675.0%) of the patients who obtained extreme scores (either the highest score or the lowest score) on one scale did not obtain extreme scores on the other scale. Last, based on the aforementioned definitions, theoretically, each of the three ADL constructs has unique characteristics and has its own value and meaning, thus making each irreplaceable [8], [10], [13], [18], [35], [36].Our results indicate that the three scales assess three unique constructs, which should be distinguished in clinical practice and research [13].

Mode of administration can have a substantial effect on the results of ADL assessments [5], [37]. The BI assesses patients’ actual performance in real life and is commonly assessed through face-to-face interview, which is easy and fast to administer [38]. However, self-reports by the patient and/or the patient’s primary caregiver might overestimate or underestimate the patient’s actual performance, and thus may affect the results of ADL assessment [5], [39]. In such cases, it is important to measure patients’ ADL function along with an objective measure (i.e., the Ability scale) to provide concrete information about what the patient can and cannot do on the tasks. Although the face-to-face interview has its own weakness, it is useful for assessing subjective feelings of difficulty (i.e., what the Self-perceived Difficulty Scale assesses) in performing ADL, as the level of difficulty is known only to the patient him/herself [10], [40]. The effects of modes of administration may affect the results of the ADL assessments; thus, it is important to use the most appropriate mode of administration to assess each construct of ADL [5].

The BI-SS is concise and quick to administer. The Self-perceived Difficulty Scale consisted of 10 items, and the Ability Scale contained only 8 items. The total time for completing both scales was appropriately 15 minutes. A short and quick-to-complete measure can lessen burdens on patients and clinicians, which is an especially important consideration for patients having severe disability. Therefore, the BI-SS appears useful in improving practice and enhancing the efficiency of administration.

It is strongly suggested that the BI-SS, which adopted the items from the original BI, be used in conjunction with the original BI to facilitate comparison and comprehensively obtain every aspect of patients’ ADL functions. The Stroke Impact Scale-16 and the Physical Self-Maintenance Scale assess the constructs of self-perceived difficulty and ability, respectively [30], [41], [42]. However, it is ideal to use the same items to assess a patient’s actual performance along with self-perceived difficulty and ability because this makes comparison of these three ADL functions of patients much more straightforward [8]. The resulting information could be useful for clinical reasoning and patient management, which may result in better treatment outcomes. In addition, using the BI-SS and the BI together can provide comprehensive (including different aspects of ADL functions) information that is useful for researchers in examining the impacts of stroke.

The subjective feeling of difficulty in performing ADL might vary substantially between persons of different ethnicities. In addition, the tools/materials used for assessing the items of feeding, grooming, dressing, and bathing in the Ability Scale may be culture-specific. Particularly, chopsticks are the most common eating utensil in Taiwan and other Asian countries. However, chopsticks are less commonly used in North America and Europe. Thus, there is a need to cross-validate our results and use culture-specific items for different countries.

Three limitations of this study are addressed. First, we excluded patients with stroke who had cognitive impairment. It was determined that patients with cognitive impairment could not report their perceived difficulty on performing ADL and could not understand instructions to perform ADL. In addition, we also excluded patients with stroke who had co-morbidities such as dementia, Parkinsonism, limb amputation, or spinal cord injury. Thus, caution should be exercised in generalizing our findings to all stroke populations. Second, the reliability between raters has not yet been established, which may jeopardize our current validation of the BI-SS. Future studies are needed to examine the reliability of the BI-SS in patients with stroke. Third, the MID of the BI (i.e., 1.85 points) was used to act as a threshold to determine whether the patients’ difference on the BI-SS had reached the MID. However, the cutoff value for the BI-SS may be different from that of the BI. The current results might be confounded by using the 1.85 cutoff as a marker for the difference in scores. Future studies to estimate the MID of the BI-SS are needed to further validate our results.

## Conclusion

The BI-SS was developed from the BI as supplementary scales in order to comprehensively assess ADL functions. The BI-SS had overall good construct validity in patients with stroke. The BI-SS could be a useful tool for assessing patients’ ADL functions and identifying patients’ difficulties in performing ADL tasks, planning intervention strategies, and assessing outcomes.

## Supporting Information

File S1 **Appendices.** Appendix S1. Items and response categories of the Barthel Index (BI) and the BI-based Supplementary Scales (BI-SS). Appendix S2. A comparison of the ADL construct and characteristics of the Barthel Index, Self-perceived Difficulty Scale, and Ability Scale.
(DOCX)
