# Supplementary material for: Development of Two Barthel Index-Based Supplementary Scales for Patients with Stroke
Source: PLoS One. 2014 Oct 20;9(10):e110494. doi: 10.1371/journal.pone.0110494 (PMC4203801; doi:10.1371/journal.pone.0110494)
Supplement: File S1 — Appendices. Appendix S1. Items and response categories of the Barthel Index (BI) and the BI-based Supplementary Scales (BI-SS). Appendix S2. A comparison of the ADL construct and characteristics of the Barthel Index, Self-perceived Difficulty Scale, and Ability Scale. (DOCX) [file pone.0110494.s001.docx]

Appendix S1. Items and response categories of the Barthel Index (BI) and the BI-based Supplementary Scales (BI-SS)

| **Item** | | **BI** | **Self-perceived Difficulty Scale**^*^ | **Ability Scale**^†^ |
| --- | --- | --- | --- | --- |
|  |  | 2 Independent | 2 Without any difficulty | 2 Able to complete^‡^ |
| 1 | Feeding | 1 needs help | 1 With some difficulty | 1 Able to complete partially |
|  |  | 0 Dependent | 0 With much difficulty | 0 Unable to perform |
| 2 | Grooming | 1 0 | 2 1 0 | 2 1 0 |
| 3 | Dressing | 2 1 0 | 2 1 0 | 2 1 0 |
| 4 | Bathing | 1 0 | 2 1 0 | 2 1 0 |
| 5 | Bowels^‡^ | 2 1 0 | 2 1 0 |  |
| 6 | Bladder^‡^ | 2 1 0 | 2 1 0 |  |
| 7 | Toilet use | 2 1 0 | 2 1 0 | 2 1 0 |
| 8 | Transfer | 3 2 1 0 | 2 1 0 | 3 Able to complete  　2 Almost able to complete  　　1 Barely able to complete  　　　0 Unable to perform |
| 9 | Mobility | 3 2 1 0 | 2 1 0 | 3 2 1 0 |
| 10 | Stairs | 2 1 0 | 2 1 0 | 2 1 0 |
| **Total score** | | 0 ~ 20 | 0 ~ 20 | 1. ~ 18 |

^*^The Self-perceived Difficulty Scale was administered to the patients via face-to-face interview. The patient was asked, “How much difficulty (without any difficulty, with some difficulty, with much difficulty) do you have in performing the (task)?” Because self-perceived difficulty is based on a patient’s own perception, it is valid only if the responses are from the patient him/herself.

^†^The Ability Scale was administered by direct observation of a patient doing each of the 8 items in an assessment room. The equipment (including chopsticks, spoons, a bowl, a brush, toothpaste, clothes, and towels) was needed for assessing the items of feeding, grooming, dressing, and bathing in the Ability Scale.

^‡^The Ability Scale scoring criteria:

For a 3-point scale:

2= Patient is able to do all the parts of the task.

1= Patient is able to do one or more parts but not all of the parts of the task.

0= Patient is unable to do any parts of the task.

For a 4-point scale:

3= Patient is able to do all steps of the task.

2= Patient is able to do more than 3 steps but not all of the steps required to

complete the task.

1= Patient is able to do one or two of the steps required to complete the task.

0= Patient is unable to do any steps of the task.

^§^Items (Bowels and bladder control) were not included in the Ability Scale due to their infeasibility and non-practicality to be assessed in clinical settings.

Appendix S2. A comparison of the ADL construct and characteristics of the Barthel Index, Self-perceived Difficulty Scale, and Ability Scale

| **Characteristic** | **Measure** | | |
| --- | --- | --- | --- |
|  | Barthel Index | Self-perceived Difficulty Scale | Ability Scale |
| **Construct** | Actual performance | Self-perceived difficulty | Ability |
| **Description** | A patient actually does an ADL in his/her daily context | A patient’s subjective perception about the level of difficulty in performing ADL without assistance | A patient’s ability to execute an ADL in a standardized, controlled context |
| **The commonly used mode of administration** | Face-to-face interview | Face-to-face interview | Observation |
| **Clinical and research application** | Useful for identifying a patient’s level of dependence/disability in real life | Useful for identifying areas of difficulty in ADL performance on the basis of patient’s reports | Useful for identifying problems in performing ADL, and thus useful for intervention |
|  | Indicating level of assistance in performing ADL tasks | Identifying unmet needs in individual patient and planning intervention toward patient-centered care | Reflecting the highest probable level of ADL functioning that a patient may reach in an assessment room without environmental distractors |
|  | Outcome indicator | Outcome indicator | Not suggested as an outcome indicator because the testing results may not be generalized to real life disability |

ADL: activities of daily living
